# Modern Digital Query Analytics of Patient Education Materials on Acanthosis Nigricans: Systematic Search and Content Analysis

**DOI:** 10.2196/60210

**Published:** 2025-01-06

**Authors:** Kevin Johny Varghese, Som Singh, Emily Kamali, Fahad Qureshi, Aleena Jamal, Fawad Qureshi

**Affiliations:** 1 Division of Dermatology, University of Kansas Medical Center, 2060 W 39th Ave, Kansas City, KS, 66103, United States, 1 8164041000; 2 Department of Biomedical Sciences, University of Missouri-Kansas City School of Medicine, Kansas City, MO, United States; 3 Department of Interventional Radiology, Loma Linda University, Loma Linda, CA, United States; 4 Department of Internal Medicine, Sidney Kimmel Medical College, Philadelphia, PA, United States; 5 Division of Nephrology and Hypertension, Department of Medicine, Mayo Clinic, Rochester, MN, United States

**Keywords:** acanthosis nigricans, dermatology, patient education, public health, skin, readability, information resource, DISCERN, general public, reading level, information seeking, information behavior

## Abstract

**Background:**

Online digital materials are integral to patient education and health care outcomes in dermatology. Acanthosis nigricans (AN) is a common condition, often associated with underlying diseases such as insulin resistance. Patients frequently search the internet for information related to this cutaneous finding. To our knowledge, the quality of online educational materials for AN has not been systematically examined.

**Objective:**

The primary objective of this study was to profile the readability and quality of the content of publicly available digital educational materials on AN and identify questions frequently asked by patients.

**Methods:**

This study analyzed publicly available internet sources to identify the most frequent questions searched by patients regarding AN using the Google Rankbrain algorithm. Furthermore, available articles on AN were evaluated for quality and reading level using metrics such as the Brief DISCERN score, and readability was determined using three specific scales including the Flesch-Kincaid score, Gunning Fog index, and the Coleman-Liau index, based on literature.

**Results:**

Patients most frequently accessed facts on AN from government sources, which comprised 30% (n=15) of the analyzed sources. The available articles did not meet quality standards and were at a reading level not appropriate for the general public. The majority of articles (n=29/50, 58%) had substandard Brief DISCERN scores, failing to meet the criteria for *good quality*.

**Conclusions:**

Clinicians should be aware of the paucity of valuable online educational material on AN and educate their patients accordingly.

## Introduction

Online digital materials are increasingly central to patient education [1]. Internet-based resources such as websites, telehealth platforms, and mobile health apps, are tools that patients interact with, in order develop health care literacy [2-4]. Furthermore, health care literacy is associated with outcomes which determine the patient’s experience [5]. Patients with poor health literacy are more likely to suffer suboptimal health care outcomes [6,7]. Digital patient education plays an important role in improving outcomes in fields such as dermatology [8].

Acanthosis nigricans (AN) is a common cutaneous disorder characterized by symmetric and velvety hyperpigmented plaques [9]. These are often found in intertriginous areas such as the posterior neck, axillae, or the inguinal or inframammary regions [9]. The prevalence of AN is as high as 74% in some populations, and its incidence increases with age [10]. Obese individuals are at higher risk for AN [10]. Furthermore, a higher prevalence of AN is observed among the Native American, African American, and Hispanic populations [11]. AN typically indicates underlying insulin resistance or other endocrinological pathologies, including malignancy [12,13]. It may also be associated with other findings such as metabolic syndrome, acrochordons, hyperandrogenism, or diabetes mellitus [14]. The characteristic hyperpigmented plaques occur due to increased levels of insulin and insulin-like growth factor 1, which stimulate keratinocyte proliferation [14]. Rarely, AN may be induced by drugs such as nicotinic acid or insulin [15]. Furthermore, some cases of AN are inherited through familial mutations in genes such as fibroblast growth factor receptor 3 (FGFR3) [16,17]. Lastly, AN can be a paraneoplastic manifestation of malignancies such as gastric adenocarcinoma [18].

Given that AN may be a manifestation of highly prevalent cardiovascular conditions such as diabetes and insulin resistance, we sought to evaluate the current online resources available to patients [12]. Additionally, patients searching online resources may encounter associations with diseases such as inherited mutations or malignancy. To our knowledge, the quality of online educational materials for AN has not been evaluated. The primary objective of this study was to assess the readability and quality of the content of publicly available digital educational materials on AN and determine the most frequently searched questions by patients.

## Methods

### Study design

In March 2024, a digital search was performed to extract 50 unique frequently asked questions on AN generated by the Google Rankbrain algorithm. The reviewers evaluated only materials in English. To reduce the impact of tracking cookies associated with the digital search, this search was performed using a newly installed internet browser. The digital articles associated with each question were examined for further health literacy analysis.The questions and digital articles that were extracted were then reviewed by 3 reviewers based on specific inclusion criteria as follows: (1) the article pertained to AN, (2) the article was publicly available without the requirement of a paid subscription, and (3) the content of the article consisted of at least 150 words and was written in English.

The extracted questions underwent evaluation using Rothwell’s classification of questions and were categorized as either fact, policy, or value. Questions were then sorted according to their category [19-22]. For each digital article associated with a question, reviewers initially categorized each article’s source as one of the following: academic institution, commercial, medical practice, government source, media outlet, or other. Following source classification, the reviewers of this study subsequently evaluated the content of each article for quality using the Brief DISCERN score [23]. The cutoff score for this instrument was established as ≥16 out of 30 for a digital article to be considered good-quality content [23]. Following quality assessment, the text of each digital article was extracted onto a plain text document and evaluated for readability. Moreover, readability was determined using 3 specific scales based on prior literature: Flesch-Kincaid score, Gunning Fog index, and the Coleman-Liau index [24-27]. This study established grade reading level recommendations for content to be approximately at the 6th-grade reading level based on previous literature [28].

### Ethical Considerations

This study did not involve human subjects, and according to University of Missouri-Kansas City Institutional Review Board, under one of the categories identified in 45 CFR 46.101 (b)(4), simple observational studies of public behavior that do not involve human subjects are exempt from institutional board approval since there is no intervention involved and the behavior is not private. The data was both anonymized and deidentified.

## Results

Among the 50 questions and associated digital articles extracted for this study, 15 (30%) of the sources originated from the government, followed by 13 (26%) from academic sources, 11 (22%) from commercial sources, and 5 (10%) from media outlets (Multimedia Appendix 1). Most questions (n=27, 54%) were classified as *fact* using Rothwell’s classification of questions. This was followed by *policy* (n=14, 28%) and *value* (n=9, 18%) (Multimedia Appendix 2).

**Figure 1.**
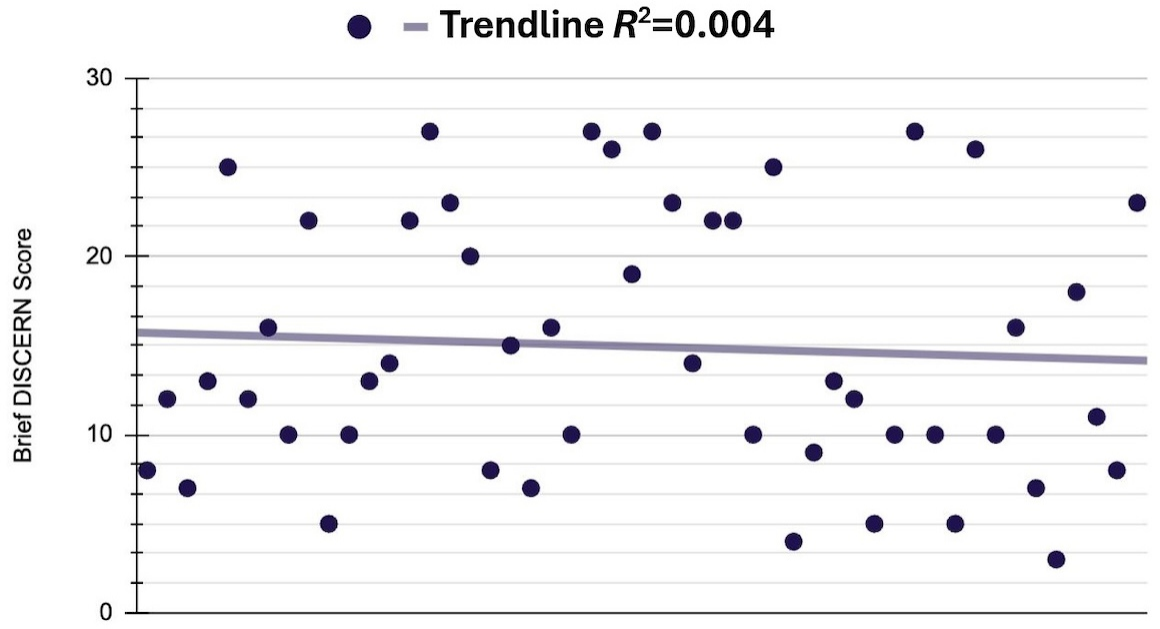
Distribution of articles on acanthosis nigricans based on the Brief DISCERN scores

The mean readability of digital articles on AN did not meet grade reading level recommendations across all 3 readability metrics (Table 1). The mean Flesch-Kincaid score of the digital articles was 11.0 (SD 3.4; range 1.7-18.2). The mean Gunning Fog score was 14.7 (SD 3.5; range 7.4-21.4). and the mean Coleman-Liau index was 13.1 (SD 3.5; range 6.0-26.7). Brief DISCERN scores for articles included in this study did not meet the recommended criteria (≥16), to be considered *good quality*; the mean brief DISCERN score was 14.9 (SD 7.3; range 3.0-27). Additionally, most (29/50; 58%) articles were substandard and did not meet *good quality* (Figure 1).

**Table 1.** Mean readability scores of available articles on acanthosis nigricans.

Variable	Tools used for analysis		
	Flesch-Kincaid score	Gunning Fog score	Coleman-Liau index
Readability scores, mean (SD)	11.0 (3.4)	14.7 (3.6)	13.1 (3.5)

## Discussion

There is a paucity of literature exploring innovations in patient education on AN [29,30]. To the best of our knowledge, this study is among the first to evaluate the quality of digital educational materials for AN using the Brief DISCERN score. Government sources emerged as the most frequent contributors to public digital education materials. The findings of this study effectively demonstrate that most articles on AN do not meet established quality standards. Furthermore, the mean readability and grade reading level of these articles are often more complex than recommended guidelines [31]. This suggests a need for improvements in the publicly available digital resources.

In addition, most of the frequently searched questions by patients on AN were classified as *facts*. his suggested that patients are investigating on AN and building their knowledge base. Furthermore, it may indicate that patients require further education from their health care providers regarding this diagnosis, as they will encounter online materials of varying quality. For example, providers should consider incorporating standardized AN educational material into clinic check-out or discharge paperwork.

A key strength of this study is the use of standardized content quality assessment tools, including readability and Brief DISCERN [23]. Readability is a well-established concept in patient education that provides a metric for reviewing educational materials qualitatively based on word count, syllables, sentence structure, etc [32,33]. This is particularly important in the field of dermatology, where prior research has found that most patient educational materials do not meet recommended reading guidelines [34]. Readability has been evaluated in literature on dermatology over the years, and most articles are at a recommended grade reading level.

However, readability alone does not appropriately and effectively evaluate content quality. As a result, this study also employed the Brief DISCERN tool. Our findings indicate that the mean Brief DISCERN score of the available articles is below the minimum quality threshold. The Brief DISCERN tool provides high reliability when evaluating online articles [23]. Investigations in the future should re-evaluate the Brief DISCERN scores of articles on AN to determine the potential effect of changes over time on the quality of these materials. A key limitation of our study is the subjective nature of Rothwell’s classification of questions, which may introduce potential bias in raters’ scoring [35,36].

In conclusion, AN is often associated with chronic diseases, such as insulin resistance, which significantly contribute to morbidity and mortality. Patients with AN frequently use the internet for education purposes. This study implemented tools established in the literature to analyze the quality, readability, and content of patient educational materials on AN. The overall quality of these materials is poor and did not meet recommended readability standards in the United States. Clinicians can improve patient outcomes by educating patients directly regarding thi condition. Consequently, patients will have access to reliable sources of information. Future studies should examine whether the quality of online materials changes over time.

## Supplementary material

10.2196/60210 Multimedia Appendix 1 Source distribution of articles.

10.2196/60210 Multimedia Appendix 2 Rothwell’s classification of questions.

## Abbreviations

AN: acanthosis nigricans
