# Supplementary figures and images for: Modern Digital Query Analytics of Patient Education Materials on Acanthosis Nigricans: Systematic Search and Content Analysis
Source: JMIR Dermatol. 2025 Jan 6;8:e60210. doi: 10.2196/60210 (PMC11728197; doi:10.2196/60210)

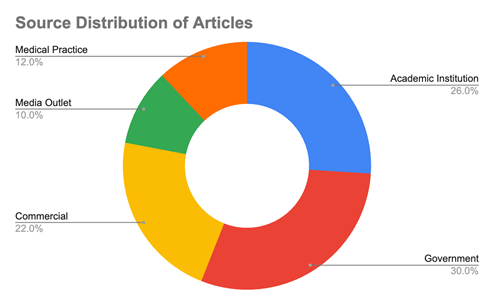

Supplement: Multimedia Appendix 1 [file derma-v8-e60210-s001.png]

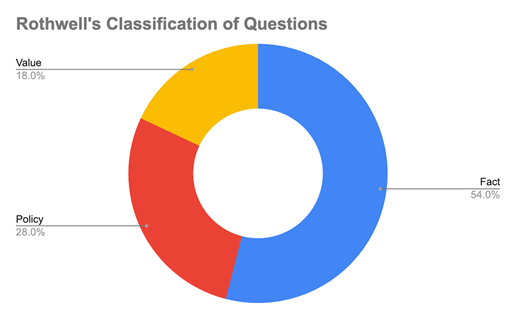

Supplement: Multimedia Appendix 2 [file derma-v8-e60210-s002.png]
